# Impact of the covid-19 pandemic on Moroccan health workers who were not on the frontline

**DOI:** 10.1192/j.eurpsy.2022.1360

**Published:** 2022-09-01

**Authors:** K. Benallel, D. Nzamba Nzamba, R. Benjelloun, M. Gartoum, M. Kadiri

**Affiliations:** 1 Military Hospital of Instruction Mohammed V, Psychiatry, Rabat, Morocco; 2 Cheikh Khalifa International University Hospital, Mohammed VI University of Health Sciences, , Psychiatry, casablanca, Morocco

**Keywords:** Impact of COVID19, Health worker, Quality of Life, DAS Scale-21

## Abstract

**Introduction:**

Since March2019, successive waves of the COVID-19pandemic have challenged the mental health of frontline healthcare workers, who have been shown in numerous studies to be at increased risk for characterized psychiatric disorders. However, healthcare professionals who were not directly involved in the care of patients withCOVID-19 were often not investigated. In Morocco, the literature dealing with the psychological impact of the pandemic on backline health professionals is still scarce.

**Objectives:**

The purpose of our survey is to assess the stress, anxiety, and depression of health workers who were not on the front lines of care forCOVID-19 patients.

**Methods:**

It is a survey developed on googleforms and disseminated via social networks targeting groups of health professionals in Morocco, during June2020. We used the DASS21 scale(depression, anxiety and stress scale with21 items). The results were analyzed using the JAMOVI application.

**Results:**

out of80 participants: 54%are men, 56%are aged between 31 and 40years old, 38%have been in practice for less than 5years of exercise, 66%are married, 41%are specialists 36%are residents and 21%are nurses, 44% work in psychiatric ward. Psychiatric symptoms: 64%have worries, 64% tired, 63% irritability, 59% sadness, 53% insomnia and 51% somatic complaints. 6.3%report substance use. DASS-21: 50%of participants have depression (7.5%mild, 26.25%moderate, 11.25%severe, 5%extremely severe), 48.75%have Anxiety (7.5%mild, 25%moderate, 8.25%severe, 7.5%extremely severe) and 30%report Stress (8.75%mild, 15%moderate, 6.25%severe, 0%extremely severe).

**Conclusions:**

The high level of job stress during the early months of the pandemic exposed health workers who were not on the front line of care forCOVID-19 patients to an increased risk of psychological decompensation.

**Disclosure:**

No significant relationships.

